# Exercise-Based Strategies to Prevent Muscle Injury in Male Elite Footballers: An Expert-Led Delphi Survey of 21 Practitioners Belonging to 18 Teams from the Big-5 European Leagues

**DOI:** 10.1007/s40279-020-01315-7

**Published:** 2020-07-16

**Authors:** Alan McCall, R. Pruna, Nick Van der Horst, G. Dupont, M. Buchheit, A. J. Coutts, F. M. Impellizzeri, M. Fanchini, the EFP-Group, Andrea Azzalin, Andreas Beck, Andrea Belli, Martin Buchheit, Gregory Dupont, Maurizio Fanchini, Duccio Ferrari-Bravo, Shad Forsythe, Marcello Iaia, Yann-Benjamin Kugel, Imanol Martin, Samuele Melotto, Jordan Milsom, Darcy Norman, Edu Pons, Stefano Rapetti, Bernardo Requena, Roberto Sassi, Andreas Schlumberger, Tony Strudwick, Agostino Tibaudi

**Affiliations:** 1 Arsenal Performance and Research Team, Arsenal Football Club, Colney, London, AL2 1DR UK; 2 School of Applied Sciences, Edinburgh Napier University, Edinburgh, UK; 3 FC Barcelona Medical Services, Ciutat Esportiva, Barcelona, Spain; 4 FIFA Medical Centre, Royal Netherlands Football Association (KNVB), Zeist, The Netherlands; 5 Football Exchange, Liverpool John Moores University, Liverpool, UK; 6 Performance Department, Paris St Germain, Paris, France; 7 Sport and Exercise Discipline Group, University of Technology Sydney, Sydney, Australia; 8 Performance Department, AS Roma Football Club, Rome, Italy; 9 Department of Neuroscience, Biomedicine and Movement Sciences, University of Verona, Verona, Italy

## Abstract

**Purpose:**

To define based on expert opinion and practical experience using a systematic and scientific approach, (1) the perceived most effective exercise-based strategies to prevent muscle injury in elite footballers; and, (2) when and how these exercise programs are prescribed based on the number of days between games i.e. implementation strategy.

**Methods:**

A Delphi survey obtained opinions and assessed for agreement. Delphi respondents consisted of 21 experienced sports practitioners (12 ± 5.3 years in elite football and with an academic background) belonging to 18 teams from the Big-5 European football leagues; England, France, Germany, Italy, Spain. Three teams were represented collaboratively by two experts. The Delphi process involves sequential rounds each evolving based on the responses from the previous. The number of rounds is not pre-defined and continues until an agreement is either achieved or it is clear that no agreement will be reached. Frequency of responses was recorded where the agreement was sought (i.e. in closed questions) and an agreement was achieved if ≥ 13/18 (70%) respondents agreed. For open-ended questions, a qualitative content analysis was performed to identify recurring themes and when themes were specified by ≥ 13 (70%), these were also considered as reaching an agreement. Practitioners had the opportunity to raise concerns if they disagreed with the ‘agreement from recurrent themes’.

**Results:**

There were four Delphi rounds (100% response for each round). Sprinting and High-Speed Running (HSR) focused exercises were agreed as most effective (perceived) to prevent muscle injuries. Eccentric exercise was perceived as the next most effective. It was agreed that sprinting and HSR be integrated into coaches training, and target 100% of players worst-case match scenario (e.g. volume, intensity) based on individual maximum speeds. Eccentric exercise was recommended to be implemented according to the context of the main football session and planned/actual sprinting and HSR content. It was agreed that eccentrics can be performed before or after training, context dependent. The day to perform specific sprinting and HSR or eccentric exercises depended on the proximity of previous and upcoming matches. Other exercises reaching agreement as ‘somewhat effective’ included concentric and isometric, horizontal and vertical plyometrics, coordination, core and dynamic flexibility in addition to core stability. No agreement was reached for multi-joint, resisted sprinting, kicking or agility exercises nor simultaneous single-leg strength and stability. Finally, no agreement was reached regarding programming variables e.g. sets, repetitions as deemed too contextual.

**Conclusion:**

Regarding exercise-based strategies, particular importance agreed by the Delphi expert group was to focus on sprinting, HSR and eccentric exercises, integrated with a variety of other exercise modes which also carry some level of effectiveness in a multidimensional programme. Context was agreed to be key and decision-making about when to undertake/ how to prescribe exercise strategies to be made according to the content of normal football training and the proximity of matches.

## Key Points

**Table:** 

A specific focus on Sprinting and High-Speed Running and eccentric exercise was perceived as being particularly important in the multidimensional exercise preventive programme.
The multidimensional exercise preventive programme appears to be extensive with 13 other exercise modes deemed as somewhat effective and should be considered when designing preventive programmes.
Exercise-based strategies were perceived as being most appropriately prescribed depending on the proximity of the preceding and upcoming matches as well as the content of the coaches training sessions.
Exercise-based preventive strategies were agreed to be maximised when combined with non-exercise strategies in a global preventive effort.

## Introduction

Injuries can have a negative impact in male elite football teams with a lower injury incidence having been correlated with elite European teams’ final league ranking [1] and an estimated average cost of one player being missing for one month due to injury of €500,000 [2]. In particular, muscle injuries, are the most common injury affecting this population. Preventing muscle injuries is, therefore, a key target of the science and medicine support staff of elite teams. Preventive strategies are widely accepted within the science and medicine support staff of elite male teams as needing to be multi-dimensional focusing on risk factors focussing on assumed risk factors for muscle injuries, of which exercise-based preventive strategies are commonly implemented with this belief [3–5]. There has been attention drawn in the research literature [6] and various social media criticising male elite science and medical teams that they are not following best evidence recommendations for exercise preventive strategies, in particular, in relation to eccentric exercise. While we agree that the gold standard approach for which exercise-based preventive strategies to use and how to implement them is with an ‘evidence-based’ process [7] i.e. a combination of high-quality scientific evidence with practical experience, no such high-quality studies have yet been conducted in male elite football.

Despite several original scientific studies supporting the use of exercise strategies to prevent muscle injuries in elite footballers, including level 1 evidence randomised-controlled trials (RCT’s) and non-randomised studies, the certainty in this evidence is not clear given the potential biases present in the original studies. Although a recent systematic review [8] recommends that by implementing the Nordic hamstring (eccentric) exercise in a prevention programme, injuries can be halved, that review integrates findings from a variety of athletic populations (different genders, ages, levels and sports), therefore the generalisability to any one specific population may be limited. Additionally, using estimates for multi-component prevention programmes e.g. the 11 + prevention programme, which incorporates the Nordic hamstring exercise in combination with other exercise modes to conclude that it is effective as an isolated exercise strategy may not be appropriate. While practitioners can extrapolate evidence from other populations (e.g. sub-elite, amateur, youth etc) and contexts, this may not be applicable particularly given that adaptations of both muscle strength and architecture are likely dependent on training status [9]. Furthermore, the training structure and week will be very different between populations such as amateur, youth, senior, team sports, individual sports etc. When the current authors (current practitioners in elite male football teams) are seeking specific recommendations to use with confidence in our setting and with our players we can start with three systematic reviews aimed at the elite male football cohort [10–12]. These three reviews highlighted that the methodological quality and/or sources of bias present in the individual studies mean that the effect of exercise preventive strategies specifically for male elite footballers is currently unknown. Indeed, the most recent of the three systematic reviews by Fanchini and colleagues [10] identified 5 randomised-controlled trials (RCT’s) and 7 non-RCT studies showing that all but one of the RCT’s were at high risk of bias and all the non-RCT’s (levels 3 and 4) were classified as ‘critical’ and ‘too problematic to provide evidence using the Risk of Bias in Non-Randomised Studies of Interventions (ROBINS-I) tool.

Whilst one may want to refer to the results of studies suffering from methodological weaknesses in the absence of there being nothing better, this choice is not an evidence-based approach but, at best, a ‘weak-evidence’ based practice encompassing a substantial degree of uncertainty. Nevertheless, regardless of the quality of the evidence, it is possible, by using established procedures, to gain additional valuable insights from practitioners experience (1) to examine whether the practice is aligned with available evidence, (2) to identify common practices for which research is needed since not currently supported by evidence, and, in absence of well-conducted high-quality, low risk of bias scientific intervention studies and (3) they can provide better indications than the experience of a single individual [13]. Single individual experience is a practitioner alternative when there is no available evidence (or too weak to have reliable recommendations). As stated by Minas et al., while the expert agreement may turn out to be wrong, consensus usually provide better judgement than the individual’s judgment [13]. Elite sports team practitioners are at the frontline of sport implementing and integrating new training programmes [9]. With multiple years of experience servicing elite players and operating with real challenges of the practical setting, practitioners can provide insight into what they believe important and how they implement strategies within the context and constraints of practice.

The aim of the present study was to define, based on expert opinion and practical experience through a systematic and scientific approach (Delphi method), (1) the perceived most effective exercise-based strategies to prevent muscle injury in elite footballers; and, (2) when and how these exercise programs can be prescribed based on the number of days between games i.e. implementation strategy.

## Methods

The Delphi consensus is a practical and structured method of obtaining opinions and achieving consensus among a group of experts on any given topic [14]. Delphi experts participate anonymously through completion of sequential questionnaires that constitute different rounds, with each round refined based on the previous one [14]. Following a recent Delphi survey on Return-to-Play in football, a similar method was employed for this study [15]. For more details on this method, we direct the reader to an overview of the Delphi process and methodology [16].

### Steering Committee

The Delphi survey was created by a 4-member steering committee consisting of two sports scientists (AM and MF), one sports physiotherapist (NvdH) and one sports medicine doctor (RP) all working in elite football and with applied research experience.

### Expert Panel

Selection of expert panel was based on the steering committee’s network and knowledge of the practitioners working with European male elite football teams. This was based on some specific criteria; (1) they were primarily responsible for the overall prevention programme in the team, (2) they spoke English to a high level or fluently, (3) they had specific knowledge and experience working as a practitioner on the football field in a full-time capacity and in applied research at the highest levels of elite football. We sent an invitation via email to the heads of performance (or equivalent title/role) of twenty elite football teams asking them to participate as Delphi respondents i.e. the expert panel. Two teams did not respond to our invitation email.

The expert panel, described from herein as the *Elite Football Performance* (EFP) Group was comprised of 21 practitioners from 18 teams participated; 3 teams provided responses based on a collaborative approach of 2 practitioners [(the teams responded collaboratively at their specific request due to language restrictions (*n* = 1) and to facilitate the busy schedule of the head of performance (*n* = 2)]. Considering all 21 practitioners, together they had 12 ± 5.3 years experience working in elite football with 8 ± 5.2 in their current position and with 6 ± 3.3 working in the Union of European Football Associations (UEFA) Champions League/Europa League.

Specifically, the EFP-Group experts were currently working in elite football belonging to a team from one of the Big-5 European football leagues as defined by the International Centre for Sports Studies (CIES) Observatory (http://www.football-observatory.com/); English Premier League (× 4 clubs), French Ligue 1 (× 2), German Bundesliga (× 3), Italian Serie A (× 6) and Spanish La Liga (× 3). Despite the experts currently belonging to one team from the Big 5 European Leagues, their experience has been varied and exposed to various elite football training methodologies and cultures; 9 experts have worked in Italian Serie A, 7 in English Premier League, 5—Spanish Primera Liga, 5 German Bundesliga, 3—French Ligue 1, 2—Scottish Premier League, 2—Russian Premier League, 2—USA Major League Soccer, and 1 in each of the Qatari, Jordanian, Austrian and Australian premier leagues. Only 6 of the experts had worked in 1 country only (2 in Italian Serie A, 2 Spanish Primera Liga and 1 English Premier League).

Fifteen practitioners held tertiary level (degree) sport science, 2 in exercise physiology and 2 in medicine/physiotherapy. Seventeen held a postgraduate qualification (10 PhD’s; 8 in sport science, 2 in physiology and 7 Masters all in sport science).

### Delphi Procedure

A series of four sequential rounds was performed. Only ‘Round 1’ was prepared in advance of commencing the study because as mentioned previously, in a Delphi survey each subsequent round depends on the responses from the previous one, evolving based on the qualitative analyses. Specifically, Round 1 focussed on identifying the types of exercises that practitioners believed to be important and their perceived level of effectiveness in preventing muscle injury. Specifically, 1 closed question was implemented asking experts to rate their level of perceived effectiveness on a Likert scale (not effective-somewhat effective-effective-very effective) for 13 different exercise modes (sprinting and high-speed running, eccentric, concentric, isometric, vertical and horizontal plyometrics, activation/coordination, dynamic and static flexibility, core stability, agility, kicking and resisted sprinting). The exercises were selected by the steering committee during the planning phase of Round 1 based on their experiences as persons responsible for an injury prevention programmes in elite footballers. Additionally, an open question was provided for experts to add any additional exercises they perceived to be important and that should go into the 2nd round. As subsequent rounds cannot be pre-defined in a Delphi survey and are based on the previous rounds’ analysis and findings, the subsequent rounds in this study actually comprise part of the results and are therefore detailed in the ensuing results section. If agreement could not be reached after 2 rounds of inclusion, this was considered as ‘no consensus’ and excluded from any further rounds.

Each round comprised an online questionnaire (SurveyMonkey, California, USA). Following each round, responses were analysed by members of the steering committee, and feedback report of main findings sent to the EFP-group in addition to the subsequent round questionnaire.

Each questionnaire was completed anonymously (i.e. EFP-group members were unaware of other individual responses and only received the global results to inform the next round) and sent back to the principal investigator (AM).

The members of the EFP-Group were encouraged to expand on their answers with justifying arguments and/or reasons supporting their responses. For questions asking experts to specify their perceived effectiveness for a strategy, respondents were asked to rate based on a qualitative Likert scale using the following descriptors; Not effective (−), somewhat effective (+), effective (++) and very effective (+++). If a preventive strategy was considered effective or very effective based on consensus among the EFP-Group (as defined below), we constructed follow-up questions on that topic about how to implement in practice.

#### Data Analysis

Responses were downloaded to Microsoft excel and a content analysis was performed. Content analysis is a qualitative research approach to analyse texts and examine patterns in a replicable and systematic manner [17]. For open-ended questions and answers, a two-step analysis and interpretation as recommended by Côté et al. [18] was followed. The first step was to create tags i.e. coding of meaningful text segments to produce a set of concepts which adequately represents the information received. The second step of interpretation analysis was to create categories which involved listing and comparing the previously created tags to produce clusters of similar tags serving as an organising system. This approach was used throughout rounds two, three and four (four being the final round). If commonalities in responses were present and reached ≥ 70% this was considered as reaching agreement/consensus among the experts (agreed a priori by the EFP-Group) and that particular item was considered complete and removed from any further rounds. This threshold was chosen in line with similar Delphi surveys [15, 19, 20]. Regarding closed questions where experts were asked to rate perceived effectiveness of an exercise or strategy or to tick a box agree/disagree on any topic, the frequency of each experts selected response were recorded and converted to a percentage to determine if they met the ≥ 70% threshold set for achieving consensus. For example, 18 experts selecting ‘agree’ and 0 selecting disagree would mean 18/18 responded agree (100%). Two of the steering committee members (AM, RP) independently performed content analyses. A third investigator (NvdH) was consulted whenever there were any disagreements/ambiguity around the tagging, categorising and interpreting of the responses.

## Results

The Delphi process started on 1st December 2017 and the final round was completed 17th March 2018. In total, there were four Delphi rounds. All EFP-Group experts participated in each round (100%). As mentioned previously, only Round 1 was prepared in advance as sequential rounds evolve based on the responses from the previous one (we refer the reader back to the methods section for details on Round 1). Below we outline the focus of rounds two to four which are a result from each prior round.

Round 1 resulted in 9/13 exercises achieving consensus, with the four not achieving consensus and two new exercise modes added by the expert panel being entered into Round 2 (Table 1).

**Table 1:** Exercise-based prevention strategies for muscle injury in elite footballers and their perceived effectiveness using the Delphi survey technique

Preventative strategy	Overall rating given^a^	% (*n* =) Consensus	Round introduced	Round where consensus was achieved
Sprinting and high-speed running	+++	72 (13)	1st	1st
Eccentric focus	++	100 (18)	1st	1st
Concentric focus	+	94 (17)	1st	1st
Horizontal plyometrics	+	89 (16)	1st	1st
Vertical plyometrics	+	83 (15)	1st	1st
Isometric focus	+	83 (15)	1st	1st
Activation/coordination (e.g. sprint movement/mechanic drills)	+	83 (15)	1st	1st
Dynamic flexibility	+	78 (14)	1st	1st
Core stability	+	78 (14)	1st	1st
Static flexibility	+	78 (14)	1st	2nd
Multi-joint exercises (e.g. olympic lifting, squats, functional strength exercises)	Between + to +++	No consensus^a^	2nd	No consensus after 2nd round
Simultaneous training of single-leg strength and stability	Between + to +++	No consensus^a^	2nd	No consensus after 2nd round
Agility	Between + to +++	No consensus^a^	1st	No consensus after 2nd round
Kicking (shooting, hard, long passes)	Between − to +++	No consensus^a^	1st	No consensus after 2nd round
Resisted sprints (e.g. sled, parachute)	Between + to +++	No consensus^a^	1st	No consensus after 2nd round

Key: (+++) very effective, (++) effective, (+) somewhat effective, (−) not effective

Multi-joint exercises—0/8/4/6

Single leg strength and stability—0/7/7/4

Agility—0/6/5/6 (1 person did not specify)

Kicking—2/11/4/0 (1 person did not specify)

Resisted sprints—2/10/4/1 (1 person did not specify)

^a^Specific responses (*n* =) for those not finding consensus based on likert scale (not effective/somewhat effective/effective/very effective)

Round 2 was targeted at finding consensus on exercise types which did not reach consensus in round 1 (resulting in 2 × closed questions). In round 1, the experts agreed (see next section for details, but we provide briefly here also, to provide context in interpreting this section) on the perceived effectiveness of 10/13 exercises proposed to them. Additionally, reasons for their perceived level of effectiveness was introduced (3 × open questions) to obtain deeper information and how to integrate the perceived most effective exercise strategies into the overall football program (3 × closed questions). One final question (closed) was added based on qualitative analysis of Round 1 to find consensus on non-exercise-based strategies. This was deemed important by the steering committee as it was a major comment in the Round 1 open ended question.

Round 3 aimed to delve deeper into methods of integrating both sprinting and HSR (including 8 × closed questions + open-ended option for to provide further details) and eccentric exercise (8 × closed + open-ended option for additional information) as these were rated as the most effective exercises to prevent muscle injury.

Round 4 represented the final round and the objective was to ‘round up’ the process. In this round we sought to find agreement on three final points in the Delphi; (1) the minimum threshold to achieve during a typical football training week for sprinting and HSR (1 × closed question), (2) performing sprinting and HSR around the same session as the main eccentric exercise session (1 × closed) and (3) optimal programming variables (sets, reps, number of exercises) for eccentric exercise (2 × open ended). At the end of each round, an open text box was provided for any details that experts felt necessary to highlight.

### Most Effective Exercise-Based Strategies to Prevent Muscle Injury

The perceived effectiveness of exercise-based strategies to prevent lower-limb muscle injuries are shown in Table 1. Sprinting (running speeds > 25.1 km/h) and High-Speed Running (HSR) (> 19.8 km/h) were perceived “very effective” with a rating of +++, achieving 72% consensus. The speeds were chosen as they are the commonly used thresholds in elite football and scientific studies. Exercises with an eccentric focus were considered “effective” (++), and all (100%) experts agreed. Eight other exercise modes were agreed as “somewhat effective”, while five could not be agreed, and were reported between ‘not effective to very effective’ (see Table 1).

### Sprinting and High-Speed Running as a Preventative Strategy for Muscle Injury

Eight of nine aspects concerning programming of sprinting and HSR exercise reached consensus while one did not (Table 2). Specifically, it was agreed that during periods of ≥ 5 days recovery between games, the preferred day to perform sprinting and HSR focussed exercise is on M − 3 and to target during the period between matches, 100% of individual players’ sprinting and HSR worst-case match scenario, however, there was no agreement if there was a minimally accepted target. During periods with ≤ 4 days recovery, no specific sprinting and HSR focused exercise was deemed necessary as the targets will likely be met during matches. There was consensus that the prescription and monitoring of sprinting and HSR loads should be performed using players’ individual maximum speeds. Finally, it was agreed that substitutes/non-playing squad players should be prescribed additional sprinting and HSR exercise on either M + 1 or M + 2, but not both.

**Table 2:** Statements/questions posed to EFP-Group regarding Sprinting and HSR and corresponding consensus/no consensus achieved

Statement (based on content analysis from each round)	Number of respondents in the final agreement (%)	Number of respondents who did not agree (%)	Round introduced	Round where consensus was achieved	Specific comments from the EFP-Group
Sprinting and HSR targeted training should be based on a players’ ‘individual’ max speeds rather than the factory set absolute thresholds	16 (94)	1 (6)	3rd	3rd	17/18 responded
Sprinting and HSR training should ideally and where possible, be integrated into the coaches’ football training drills	16 (89)	2 (11)	3rd	3rd	No specific comments
It is appropriate to use specially designed football drills and/or generic running drills to ensure Sprinting and HSR targets are met	18 (100)	0 (0)	3rd	3rd	No specific comments
Concerning starters, in a week with 6 full days recovery between matches (e.g. Saturday to Saturday) the most appropriate day to perform specific Sprinting and HSR is on Matchday − 3 (M + 4)	14 (78)	1 (6)	2nd	3rd	3 (17%) respondents neither agreed nor disagreed While the majority agreed this is appropriate, 4 experts within the ‘yes’ group Highlighted that while they agreed, there were also other options such as M − 4
Concerning starters, in a week with 5 full days recovery between matches, M − 3 (M + 3) is the most appropriate day to perform sprinting and HSR exercise	14 (78)	0 (0)	2nd	3rd	4 Experts neither agreed nor disagreed—they specified that it is an option, but also other times may be suitable and can be dependent on a number of factors including the coaches plan for sessions, whether single or double sessions will be performed
Concerning starters, in a week with ≥ 5 days recovery between matches, the ideal target of Sprinting and HSR GPS metrics to reach is 100% of the player’s worst-case match scenario	12 (71)	5 (29)	2nd	3rd	17 responses included in the analysis 1 respondent stated not using a specific % threshold
Concerning starters, during weeks with ≤ 4 days recovery it is not necessary to perform any targeted Sprinting and HSR exercise. The reason given was that players should reach their individual targets during matches (if playing full to most of the matches)	16 (89)	1 (6)	2nd	3rd	1 (6%) neither agreed nor disagreed—it was specified that players do not always reach their maximums in matches and should be monitored carefully and adjust the week plan accordingly
Non-starters/non-playing squad players should be prescribed additional Sprinting and HSR exercises on either M + 1 or M + 2 (but not both)	14 (78)	1 (6)	2nd	3rd	10 (56%) also specified that after the match is a possibility. It depends on the training and match schedule for the upcoming week. 3 neither agreed nor disagreed
Concerning starters, in a week with ≥ 5 days recovery between matches, what is the minimum target of Sprinting and HSR GPS metrics to reach in regards to the player’s worst-case match scenario	See comment box for specific details on responses		2nd	No consensus after 4 rounds	Seven (41%) respondents recommended a minimum weekly target of 50–74%, 2 (12%) stated 75–84% and seven (41%) suggested 85–100%, while 1 person (6%) proposed anything > 60%

*HSR* high-speed running, *GPS* global positioning system

### Eccentric Exercise as a Preventative Strategy for Muscle Injury

Fifty percent (*n* = 4) of aspects relating to eccentric exercise programming for muscle injury prevention reached consensus (Table 3). It was agreed that during periods of ≥ 5 days recovery between games, the preferred day to perform eccentric focussed exercise is on M + 3. However, in a between-match cycle with 5 full days, this means that the eccentric exercise coincides with M − 3 and the specific sprinting and HSR session. In this instance, the EFP-Group did not agree on whether or not it is appropriate to perform both around the same session. It was agreed that eccentric exercise can be performed either before or after football training. It was agreed that low-intensity (defined as reduced volume and intensity) eccentric exercises can be performed during periods with ≤ 4 days recovery between matches, assuming players are sufficiently accustomed. No consensus was reached regarding an optimal number of eccentric exercises in any one session nor the ideal range of a number of sets and repetitions per exercise.

**Table 3:** Statements posed to EFP-Group regarding eccentric exercise and corresponding consensus/no consensus achieved

Statement (based on content analysis from each round)	Number of respondents in the final agreement (%)	Number of respondents who did not agree (%)	Round introduced	Round where consensus was achieved	Specific comments from the EFP-Group
Concerning starters					
During weeks with 6 full days recovery between matches that the most appropriate day to perform a specific session of eccentric exercises is on the Matchday + 3 (i.e. M − 4)	14 (78)	1 (6)	2nd	3rd	3 (17%) stated that it depends (neither agreeing, nor disagreeing)
During weeks with 5 full days between matches it is possible to perform eccentric exercise on the M + 3 (M − 3)	15 (83)	1 (6)	2nd	3rd	2 (11%) neither agreed nor disagreed, specifying that it depends, for example on the coaches plan for the session
Low-intensity eccentric exercises can be performed during periods with ≤ 4 days recovery	13 (71)	4 (24)	2nd	3rd	While consensus was reached, it was also clear from the responses that players should be accustomed to doing this Additionally, it was highlighted that ‘activation’ type exercises may be preferred here Those stating that they did not agree specified that; If eccentric is being performed it should be with high loads Use exercises that are less damaging 1 (6%) respondent was not familiar with the term, ‘low-intensity eccentric exercise’ and therefore was not included in analysis
Would you agree that it is possible to vary the timing of eccentric session between both before AND after training?	15 (83)	3 (17)	2nd	4th	However, it depends on the planned and actual intensity, volume and contents of the field session. It was specified that the timing of eccentric exercise according should be adjusted according to what is planned in the field session Also depends on the target of the session e.g. to improve strength
It is possible to include eccentric exercise around the same session as the main sprinting and HSR exercise	12 (67)	6 (33)	2nd	4th	While 12 experts (67%) agreed that it is possible, however, it was highly contextual depending on (1) the amount of sprinting and HSR performed/to be performed and on the planned session on the following day. It was stated by 1 respondent that a double session may be a solution for this
What are the number of eccentric exercises that should be included in any given session?	No consensus		2nd	No consensus after 4 rounds	
What is the optimal number of sets and repetitions for (a) low-intensity eccentric exercise and (b) high-intensity eccentric exercise?	No consensus		2nd	No consensus after 4 rounds	The variability of sets and reps from each respondent was very high (see Appendix 1 for individual responses). Overall, the reason given was that this is too contextual and depends on the specific exercises, when they are being performed and who is performing them

### Muscle Injury is Multifactorial and Prevention Should be Multidimensional

While the focus of the current Delphi survey was on exercise-based strategies to prevent muscle injury, the respondents also highlighted in Round 1 an interesting and pertinent consideration that they wanted to take through to Round 2. When asked ‘other strategies’ to prevent muscle injury, they agreed that a combination of strategies will maximise preventative efforts targeted at muscle injury. Specifically, consensus was achieved in Round 2 for’ (1) overall control of load/management of the training week (18/18 respondents; 100%, +++ very effective), (2) consideration of previous injury (17/18; 94%, ++ effective), (3) ability to work together (16/18; 89%, ++ effective), (4) team communication (15/18; 83%, ++ effective), (5) recovery strategies (14/18; 78%, ++ effective).

## Discussion

Our expert EFP-Group agreed that their most effectively perceived exercise-based strategies to prevent muscle injury were (see Table 1); sprinting and HSR (very effective +++), eccentric (effective ++) with horizontal and vertical plyometrics, dynamic and static flexibility, core stability, concentric and isometric rated as somewhat effective (+). Consensus was not reached on precise effectiveness of some other exercise modes; multi-joint exercises (e.g. squats, Olympic style lifts etc), single-leg strength and stability and agility were rated between somewhat effective to very effective while kicking and resisted sprints ranging between not effective through to very effective. The day to perform specific sprinting and HSR or eccentric exercises depended on the proximity of previous and upcoming matches. While the first round of our Delphi survey highlighted a number of exercise strategies as at least being somewhat effective and others with an unagreed level of perceived effectiveness, the scope of covering all exercise types (i.e. 15 exercise types were highlighted in Round 1, with 10 agreed on for their perceived level of effectiveness), both in ensuing rounds of the Delphi and in this paper are too great. As such, we focused our efforts on obtaining deeper information on the two most importantly perceived exercise types; sprinting and HSR in addition to eccentric. We would like to make it clear to the reader from the outset, that by doing so we are not negating the potential role of the others nor are we confirming that the choices of the steering committee are necessarily the correct choice. The objective of the Delphi survey is to present the perspectives and agreements of practitioners working in the “field” irrespective of their real efficacy. The Delphi experts also requested (and later found consensus on) highlighting that exercise-based strategies must be accompanied by other non-exercise-based strategies if preventive strategies are to be optimised. These were; management of training week (+++), consideration of the previous injury, ability to work together, team communication and recovery strategies (all ++).

### Sprinting and HSR

While sprinting and HSR has traditionally been viewed as a ‘problem’ (i.e. injury mechanism), there is now emerging opinion to suggest that when integrated into a well-planned programme, it may actually provide a ‘solution’ [21] i.e. preventive effect. Our finding lends some subjective support to this notion. Given the most common muscle injury in elite football is the hamstring injury [21, 22], combined with reports that the majority occur during sprinting and HSR [23, 24], it seems appropriate to suggest that sprinting and HSR has a pivotal role, though the precise role as a protective factor still needs to be confirmed and if so, then clearly defined. It is important to highlight, that high-quality scientific evidence regarding any role of sprinting and HSR in muscle injury prevention is still lacking in male elite football and currently this supposition is based only on opinion [25] (including the current Delphi survey). Accordingly, we strongly recommend these suggestions be an urgent priority to validate (or refute) by researchers working in the area of male elite football.

#### EFP-Group Agreement #1: Individualise Sprinting and HSR Based Relative to the Players’ Own Maximum Speed

It was agreed that prescription/monitoring of sprinting and HSR should be based on individuals’ maximum speeds and not on absolute thresholds as commonly defined in previous literature and often used as factory settings on GPS. This follows logic, that when using individual speed thresholds, slower players perform greater amounts of high and very high-speed running compared to faster players who perform less when compared with absolute thresholds [26]. We recommend a distinction between sprinting and HSR. Although limited evidence exists, it may be necessary to achieve maximal or near-maximal speeds, as high-speeds alone might not be sufficient to protect against injury [27–29]. However, since there are few studies examining the effect of sprint speed/exposure on injury in elite footballers, future investigations are required.

#### EFP-Group Agreement #2: Integrate Sprinting and HSR into Coaches’ Training Drills

Wherever possible and appropriate, it was agreed that sprinting and HSR exercise should be integrated into coaches normal training drills. This will likely have important implications to gain coach buy-in and enhance player motivation to perform such maximal natured actions with quality. It may also provide greater specificity of muscle actions that cannot be targeted during generic sprinting drills (e.g. sprinting and kicking ball at full speed) and concomitant decision-making. Despite a preference to incorporate sprinting and HSR into the technical/tactical drills designed by the coach, the experts agreed that it is appropriate to implement specifically designed football drills and generic running (e.g. maximal aerobic speed, repeated sprinting etc.) to ensure players are exposed to sufficient amounts of these activities. This may be particularly important to ensure maximal and near-maximal speeds are achieved as incorporating during drills such as small-sided games may not allow players to reach such speeds. It is beyond the scope of this article to go into great detail on designing individual, position-specific drills, however, we do provide some insight into the overall principles and direct the reader to some potentially useful guides by Bradley et al. [30], Buchheit [31, 32] and the FC Barcelona Muscle Injury Guide [33] for further insights and specific recommendations into practical programming. It is important to note that these are guides only and any effectiveness has not been scientifically validated, however, overall it is suggested that replicating running profiles of players is not sufficient and the types of runs must be considered also. For example, Bradley et al. [30] recommend an integrated approach based on match analysis using types of runs while your team is in possession (e.g. drive inside, run behind/the channel, overlap, break into box, push up pitch) and out of possession (e.g. close down, cover, recovery run, ball over top/down side). Buchheit [31, 32] showed how HSR can be programmed in real-life scenarios to maintain a stable week-to-week HSR load for substitutes especially (24), and how the optimal HSR dosage can be prescribed in relation to (1) the HSR demands of other tactical/technical contents and matches and (2) the different weekly microcycles length (i.e., number of days between games). The FC Barcelona Guide [33] highlights approaching training drills to target alongside the player integration of player and position-specific technical tasks (1) neuromuscular components as well as (2) metabolic conditioning, where neuromuscular training refers to accelerations, decelerations and changes of direction and metabolic conditioning referring to the contribution and development of the aerobic and/or anaerobic energy systems. Care may need to be taken when implementing maximal efforts into training drills as inappropriate inclusion could feasibly also increase the risk of injury.

#### EFP-Group Agreement #3: Players Should Accumulate 100% of Their Worst-Case Match Sprinting and HSR Over the Training Week, But We Aren’t Sure How to Define That

During periods with one match per week (≥ 5 days), it was agreed that the ideal GPS-based target for sprinting and HSR is 100% of individual players worst-case match scenario i.e. what the players maximum demands for these metrics. Viewing worst-case scenario as a global figure from a match (as was the case in this Delphi) raises some conceptual concerns (which were beyond the scope of both our Delphi survey and this article), however, it is important to acknowledge. We still do not know exactly what the worst-case scenario is in terms of volume or intensity or both, nor how it is distributed across a match. For example, a target of 200 m of sprint distance from a match may be the highest distance sprinted by a player, but how is this actually accumulated over the course of a match? For example, that 200 m in the match could (theoretical example only) be composed of 10 × 20 m efforts, so would training with 4 × 50 m efforts be appropriate to match the specific demands. Additionally, are the sprinting efforts linear or curved for instance. These are crude examples, but just to highlight that a global WCS from motion analysis data likely does not provide the full picture. Furthermore, one or two isolated GPS metrics (as highlighted here) does not consider other important football actions such as change of direction, dribbling, jumping, passing and shooting nor does GPS give an indication of internal psychophysiological load. Preparing players to perform a global worst-case scenario is not the same as preparing them to sustain repeated phases of high-intensity activities, nor coping with such activities towards the end of matches when players may be fatigued both physically and mentally. Future work is urgently needed to determine what a ‘worst-case scenario’ actually is (and how it can be defined and quantified, and even if such a phenomenon exists) for both internal and external load measures and how these can be appropriately trained.

During ≥ 5 days between matches, it was agreed that sprinting and HSR focused exercise be performed on M − 3 (i.e. 72 h prior to next match) which falls on either M + 3 or M + 4, depending on whether it corresponds to a 5 or 6 day between-match cycle. Importantly, when there are ≤ 4 days between matches, it was agreed that no specific sprinting and HSR exercise be prescribed to starters, as targets are probably attained during matches. However, substitutes should be prescribed additional sprinting and HSR exercise on Matchday + 1 (M + 1) or M + 2, (depending on scheduled rest day) but not on both days. Indeed, it has been proposed that match-play is an important stimulus (in particular neuromuscular load) to ensure players are prepared for match-play and this is necessary to replicate in substitute/non-playing squad players [34].

### Eccentric Exercise

Eccentric exercise was perceived as an ‘effective’ (++) exercise strategy for muscle injury prevention. This is consistent with perceptions of other elite football practitioners from premier league [3] European Champions League [5] and the 2014 FIFA World Cup [4]. Eccentric exercise may be particularly useful as it targets various potential and (importantly) modifiable risk factors for muscle injury [27] including, eccentric strength, optimal angle of peak torque, and muscle architecture e.g. fascicle length. It, therefore, appears that practitioners are attempting to follow evidence-based guidelines in their practice. Even if the scientific evidence in elite male players is not clear due to various sources of bias as highlighted previously, it is not typically the role of the sport scientist in a team to analyse the risk of bias in a study. Researchers should focus carefully on the design, implementation and reporting of high-quality, low-risk of bias studies.

#### EFP-Group Agreement #4: Integrating Eccentric Exercise into the Football Training Week

To our knowledge, there is no scientific evidence with regard to the optimal day/s to implement eccentric exercise in the elite football training week. According to the EFP-Group, during periods with ≥ 5 days between matches, 3 days post-match (M + 3) is the preferred day to perform the main eccentric exercise session of the week. Importantly, however, if we consider a week with 5 days recovery, M + 3 also represents the M − 3, which, in the same cycle, is the preferred day to perform sprinting and HSR exercise. When asked if it is appropriate to perform both sprinting and HSR in addition to eccentric exercise around the same session, there was no agreement. The responses revealed that this could be appropriate, but is highly contextual and depends on the planned sprinting and HSR session, and the decision should be based around this. One respondent proposed a double session (i.e. morning and afternoon) may remedy this problem. Another respondent also highlighted that eccentric exercise performed the day before a main sprinting and HSR session should consider the content of that session before implementing eccentric exercise. Overall, it appears that the context and content of preceding and upcoming sessions as described above is critical when planning preventive exercises.

During periods with ≤ 4 days between matches, it was agreed that low-intensity eccentric exercises (defined as ‘low-load, low volume) can be used. To our knowledge, there is no scientific evidence for low-intensity eccentric exercises to prevent muscle injury in elite footballers. A common theme in the written responses was that players should be accustomed to performing the eccentric exercise to allow these during congested periods.

While not in elite players, one study in semi-elite has investigated the scheduling of injury prevention exercise [35]. When eccentric exercise was performed on M + 3 (as was agreed in our Delphi) both residual fatigue (perceived muscle soreness) and muscle damage markers (creatine kinase) were present on M − 1. Importantly, however, muscle function measured by isometric muscle contraction (muscle function is considered the gold standard for assessing muscle damage and persists with the muscle until fully recovered) [36] was unaffected by scheduling eccentric exercise on M + 3 in a 6 day match cycle. Interestingly, the same study demonstrated that performing eccentric exercises on M + 1 was tolerated by players and resulted in no residual fatigue or muscle damage markers evident on the M − 1. This could be particularly important during periods ≤ 4 days between matches to maintain any detraining of muscles [35]. However, these are speculations and need to be thoroughly investigated to make confident programming decisions with male elite footballers.

#### EFP-Group Agreement #5: Eccentric Exercise can be Performed Either Before or After Football Training

A key question in the design of the eccentric exercise prevention programme is when to perform the exercises i.e. before or after training? It was agreed in our Delphi survey that it can be appropriate to perform either before or after football training. While lacking scientific evidence, preliminary studies reveal that there may be different adaptations for before or after. Performing eccentric exercise before training has resulted in fascicle length increases (short fascicle length proposed as an injury risk factor [37]) but not when performed after the session [35]. Similar chronic adaptation of peak torque production of the hamstring muscles has been shown to be similar when eccentric exercise is performed before and after the training session [35]. Conversely, when eccentric exercise is performed after the session increases in muscle thickness and pennation angle have been observed [35]. Additionally, chronic adaptation towards an improved ability of players to maintain their eccentric strength at half-time and upon cessation of a simulated football match have been found versus those performing in a fresh state before training [38]. Again, it is important to comment that the responses we received highlight that knowledge and consideration of the preceding session/s and upcoming session/s are important when deciding when is most appropriate to schedule the eccentric session. One response from an EFP-Group expert was to prioritise ‘safety’ rather than a specific adaptation i.e. to perform the eccentric training after so that players have full strength when going into the training session. Again, this represents an expert opinion and we need high-quality research to provide higher evidence-based recommendations.

#### No Agreement on Programming Variables

We did not reach an agreement regarding specific programming variables of eccentric exercise, i.e. optimal number of exercises, sets and repetitions, nor an appropriate range. Therefore, even if a specific range is defined in the scientific literature e.g. to maximise strength or hypertrophy, it appears evidence-based recommendations would be difficult to fully apply in the practical setting and will depend on a given context within the football team. “Appendix” provides the individual responses of the EFP-Group and highlights the variability in responses.

## Strengths and Limitations

Our expert-led Delphi survey has revealed some novel and potentially practical information and we would like to acknowledge some strengths and limitations to the present study. The strengths of our study include; (1) a unique collaboration of a typically difficult to source population i.e. sports practitioners operating at the highest level of European male elite football. (2) We have extended the knowledge in the area of muscle injury prevention by going beyond simply determining basic information about which exercises are perceived to be effective. By implementing the Delphi method, we have been able to delve deeper into reasons why practitioners perceive this effectiveness in addition to specific implementation strategies within real-world context. (3) We had a 100% response rate in each of the four Delphi rounds. (4) Finally, we have elaborated in more detail on a relatively new technique (at least in the sports sciences research domain) of qualitative analysis.

Despite these strengths, we also highlight several limitations to our study; (1) From starting the Delphi to publication has taken one and a half years and it is possible that the Delphi experts views could have changed in that time with additional experience and new research being published. This is unfortunately a reality of the delay between research being conducted and subsequently published. (2) The nationality of practitioners where they have gained their education and experience may introduce a specific way of thinking and practicing in regard to training methodology and our sample does not represent an equal distribution across countries. We acknowledge that this could be a limitation but equally, the combined experience of the included experts actually spanned 12 countries and 3 continents with only 6 of the 18 experts having experience in only 1 country. (3) A further limitation is that the EFP-Group consists of predominantly sport science professionals and other practitioners e.g. physiotherapists or doctors may have different opinions. Nevertheless, our Delphi experts were key members in the primary group in their team responsible for the injury prevention program and had extensive experience with this role. The opinions and practices of other practitioners had the sample not been selectively recruited based on our network, may have been different. (4) We did not extensively follow up on aspects not reaching an agreement which could have led to greater insight into the reasons for their responses. Previous personal experiences may have influenced their practice and perceptions. For example, no consensus was reached for compound exercises (e.g. weightlifting derived exercises) but we do not know whether this was due to lack of confidence in their preventive role, feasibility issues (e.g. low technical competency of players) or insufficient previous experience with these kinds of exercises. (5) Our Delphi includes only 18 teams (21 practitioners) and the insights with additional participants may have varied. (6) Our Delphi survey rounds and the core discussion of this paper is focussed on the two most importantly perceived exercises (sprinting and HSR, in addition to eccentric) and there were 11 other exercise type reaching consensus, with two others unagreed with varying levels of perceived effectiveness. A limitation of the Delphi approach and our decision to include only the two most effectively perceived exercises means that some important information on how the others can and should be incorporated into a global exercise plan have been missed. However, we deemed it necessary to narrow our focus to gain a deeper understanding of the two top-rated exercises, otherwise to gain meaningful insights from practitioners would have been impossible and the results would have ended up superficial with no real impact or meaningful addition to the research literature. (7) One of our inclusion criteria was that respondents had to speak English fluently and may reduce representativeness from those who do not have English as a first or second language. However, to include other languages would have required cross-cultural translations (i.e. forward, back translations) for each round questions and answers and was not feasible from the resources (time, finances etc) available to our group. (8) Finally, while the Delphi survey method is a scientific approach to achieving agreement among experts, it nevertheless represents level 5 evidence (expert opinion) and we strongly encourage the reader to interpret the findings within this context.

## Conclusion

Overall, the results from our Delphi survey revealed a number of exercise modes deemed to carry some level of effectiveness to prevent muscle injury in elite male footballers. A specific importance was placed by the EFP-Group on the use of sprinting and HSR as well as eccentric exercise. The implementation of exercise strategies by the EFP-Group depended on the number of matches per week in addition to the planned content of the preceding and following football sessions (e.g. what the coaches training composes of). We strongly remind the reader that although the present study was conducted using a scientific process to attain agreement/consensus on a given topic, our findings nevertheless still represent only a level 5 expert opinion (and of a relatively small cohort), these are not substantiated by any higher level of evidence. This also suggests that in practice the strategies proposed most strongly by the literature are not necessarily the priority of practitioners working in teams where programmes appear to be implemented by combining experience and personal beliefs. As such, future high-quality, low risk of bias research should aim to validate or refute the opinions and practices found in this Delphi survey to improve confidence in implementing exercise-based strategies in elite footballers.

### Data Availability Statement

The datasets generated during and/or analysed during the current study are not publicly available due to confidentiality and ensuring anonymity of responses obtained in this qualitative research project.

## Appendix

See Table 4.

**Table 4:** Table of EFP-Group responses to specific programming variables for eccentric exercise to prevent muscle injuries

Typical number of exercises in any one session	Most effective range of load	Most effective range for velocity of contraction	Most effective range of sets and repetitions
1–5	1.0–1.2 Body mass (BM)	Nordics—Slow. Plyometrics—quick	3 sets × 3–7 repetitions
4–6	4–6 repetition maximum (RM)	1 s concentric 2–4 s eccentric	12–24 repetitions total
2	+ 20 kg plate	3–5 s	2–3 sets × 6 repetitions
1–3	Depending on exercise, 1 leg Romanian Deadlift 2–3 sets × BM, Nordic hamstring (BM) or BM plus 5–10%	Eccentric implies slow…..resisting motion	2–3 sets × 3–6 repetitions
6–8 (2 × hamstring (open/closed chain), 1–2 × Quads, 1 × Add and 1 × calves)	BM + 10–20 kg	Depends on microcycle—try fast as often as possible	4 sets × 8 repetitions
1 Exercise × muscle groups	80–90% repetition maximum	High	8–10 repetitions
12–18	Isoinertial or BM	Maximal possible (max power)	1 set × 6–8 repetitions
5	Some free weight, and some with isoinertial machines. Non-isotonic machines and BM exercises	Some slow (submaximal/control), Some high speed	For each exercise; 2 sets × 6–8 repetitions (es versapulley: 2 sets × 8 repetitions)
One main pull or push and then 2 complimentary pulls or pushes	Depending on the quality trying to develop	Eccentrics are usually done at a slower rate of 3–5 s lowering	3–5 sets × 2–6 repetitions
1	Body mass	20 deg/s	1–3 sets × 3–6 repetitions
8	100% of their individual targets	High and low	2–3 sets × 6–8 repetitions for each exercise
3–6 exercises	From submaximal to maximal eccentric force production	0.2/3 to 1.5 m/s	2–3 sets per exercise × 4–5 repetitions and up to 15–20 repetitions depending the exercise and the aim
1–2	50–70% repetition maximum	Controlled	2–3 sets × 4–6 repetitions
Usually alternate depending on the movement. In hamstring: 1 or 2 hip dominant, 1 or 2 knee dominant, 1 or 2 mix. More focus on biceps femoris or semi-tendinosous. In total not more than 3–4 different exercises	Depending the exercise	Combination of fast and slow contraction	Depending. Nordic HS 2 × 4 sets or Go and Back with rope very fast 2 × 8 sets. Try to use the minimum number of sets possible that produce adaptation
1–2	Flywheel	High	3 sets × 7 reps
During the main session: 4 exercises, one for each group muscle (quadriceps, hamstrings, calf, adductors)	Using isoinertial overload machine load can be considered a number of inertial, we can rate as the medium load that allows to perform high power	Maximal	2–3 sets of 6–10 repetitions for each exercise depending on the week or individual markers of pain

*BW* bodyweight, *HS* hamstrings, *NHS* Nordic hamstring exercise

## References

[1] Hagglund M (2013). Injuries affect team performance negatively in professional football: an 11-year follow-up of the UEFA Champions League injury study. Br J Sports Med.

[2] Ekstrand J (2013). Keeping your top players on the pitch: the key to football medicine at the professional leve. Br J Sports Med.

[3] McCall A (2014). Risk factors, testing and preventative strategies for non-contact injuries in professional football: current perceptions and practices of 44 teams from various premier leagues. Br J Sports Med.

[4] McCall A (2015). Injury prevention strategies at the FIFA 2014 World Cup: perceptions and practices of the physicians from the 32 participating national teams. Br J Sports Med.

[5] McCall A, Dupont G, Ekstrand J (2016). Injury prevention strategies, coach compliance and player adherence of 33 of the UEFA Elite Club Injury Study teams: a survey of teams’ head medical officers. Br J Sports Med.

[6] Bahr R, Thorborg K, Ekstrand J (2015). Evidence-based hamstring injury prevention is not adopted by the majority of Champions League or Norwegian Premier League football teams: the Nordic Hamstring survey. Br J Sports Med.

[7] Coutts AJ (2017). Challenges in developing evidence-based practice in high-performance sport. Int J Sports Physiol Perform.

[8] van Dyk N, Behan FP, Whiteley R (2019). Including the Nordic hamstring exercise in injury prevention programmes halves the rate of hamstring injuries: a systematic review and meta-analysis of 8459 athletes. Br J Sports Med.

[9] Buchheit M (2019). Injury rate and prevention in elite football: let us first search within our own hearts. Br J Sports Med.

[10] Fanchini M, Steendahl IB, Impellizzeri FM, Pruna R, Dupont G, Coutts A, Meyer T, McCall A (2019). Exercise-based strategies to prevent muscle injury in professional footballers: Part I—A systematic review. Sports Med.

[11] McCall A (2015). Injury risk factors, screening tests and preventative strategies: a systematic review of the evidence that underpins the perceptions and practices of 44 football (soccer) teams from various premier leagues. Br J Sports Med.

[12] Michalis AH, Apostolos S (2016). Hamstring strains in football. Prevention and rehabilitation rules. Systematic review. Biol Exerc.

[13] Minas H, Jorm AF (2010). Where there is no evidence: use of expert consensus methods to fill the evidence gap in low-income countries and cultural minorities. Int J Ment Health Syst.

[14] Slade SC (2014). Standardised method for reporting exercise programmes: protocol for a modified Delphi study. BMJ Open.

[15] van der Horst N (2017). Return to play after hamstring injuries in football (soccer): a worldwide Delphi procedure regarding definition, medical criteria and decision-making. Br J Sports Med.

[16] Fink A (1984). Consensus methods: characteristics and guidelines for use. Am J Public Health.

[17] Patton M (2002). Qualitative research and evaluation methods.

[18] Côté J, Salmela J, Baria A, Russell SJ (1993). Organizing and interpreting unstructured qualitative data. Sport Psychol.

[19] Kleynen M (2014). Using a Delphi technique to seek consensus regarding definitions, descriptions and classification of terms related to implicit and explicit forms of motor learning. PLoS One.

[20] Verhagen AP (1998). The Delphi list: a criteria list for quality assessment of randomized clinical trials for conducting systematic reviews developed by Delphi consensus. J Clin Epidemiol.

[21] Edouard P, Mendiguchia J, Guex K, Lahti J, Samozino P, Morin JB. Sprinting: a potential vaccine for hamstring injury? [cited 2019 1 July 2019]. 2019. https://sportperfsci.com/sprinting-a-potential-vaccine-for-hamstring-injury/.

[22] Ekstrand J, Hagglund M, Walden M (2011). Epidemiology of muscle injuries in professional football (soccer). Am J Sports Med.

[23] Crema MD (2016). Acute hamstring injury in football players: association between anatomical location and extent of injury—a large single-center MRI report. J Sci Med Sport.

[24] Ekstrand J (2012). Hamstring muscle injuries in professional football: the correlation of MRI findings with return to play. Br J Sports Med.

[25] Mendiguchia J (2016). Field monitoring of sprinting power-force-velocity profile before, during and after hamstring injury: two case reports. J Sports Sci.

[26] Murray NB (2017). Individual and combined effects of acute and chronic running loads on injury risk in elite Australian footballers. Scand J Med Sci Sports.

[27] Butler S. Running fast: the cause, the cure and a vaccine. In B.J.o.S. Medicine, Editor. 2019, British Journal of Sports Medicine.

[28] Malone S (2017). High chronic training loads and exposure to bouts of maximal velocity running reduce injury risk in elite Gaelic football. J Sci Med Sport.

[29] Stares J (2018). Identifying high risk loading conditions for in-season injury in elite Australian football players. J Sci Med Sport.

[30] Bradley PS, Ade JD (2018). Are current physical match performance metrics in elite soccer fit for purpose or is the adoption of an integrated approach needed?. Int J Sports Physiol Perform.

[31] Buchheit M. Managing high-speed running load in professional soccer players: the benefit of high-intensity interval training supplementation. Managing high-speed running load in professional soccer players: The benefit of high-intensity interval training supplementation 2019 [cited 2019 1 July 2019]. https://sportperfsci.com/wp-content/uploads/2019/03/SPSR60_Buchheit_190308_final.pdf.

[32] Buchheit M. Programming high-speed running and mechanical work in relation to technical contents and match schedule in professional soccer. 2019 [cited 2019 7 December 2019]. https://sportperfsci.com/wp-content/uploads/2019/07/SPSR73_Buchheit_190719_final.pdf.

[33] Pruna R, Andersen T, Clarsen B, McCall A. Muscle injury guide: prevention of and return to play from muscle injuries. 1st edn. 2018: FC Barcelona: Barça Innovation Hub.

[34] Morgans R, Di Michele R, Drust B (2018). Soccer match play as an important component of the power-training stimulus in premier league players. Int J Sports Physiol Perform.

[35] Lovell R (2018). Hamstring injury prevention in soccer: before or after training?. Scand J Med Sci Sports.

[36] Warren GL, Lowe DA, Armstrong RB (1999). Measurement tools used in the study of eccentric contraction-induced injury. Sports Med.

[37] Timmins RG (2016). Short biceps femoris fascicles and eccentric knee flexor weakness increase the risk of hamstring injury in elite football (soccer): a prospective cohort study. Br J Sports Med.

[38] Small K (2009). Effect of timing of eccentric hamstring strengthening exercises during soccer training: implications for muscle fatigability. J Strength Cond Res.

